# Synthesis, X-Ray Structure, Hirshfeld Surface Analysis, DFT Calculations, and Molecular Docking Studies of Nickel(II) Complex with Thiosemicarbazone Derivative

**DOI:** 10.1155/2021/5536902

**Published:** 2021-05-26

**Authors:** Uwaisulqarni M. Osman, Sharmili Silvarajoo, Muhamad Fairus Noor Hassim, Suhana Arshad, Ainizatul Husna Anizaim, Fazira Ilyana Abdul Razak

**Affiliations:** ^1^Faculty of Science and Marine Environment, Universiti Malaysia Terengganu, 21030 Kuala Nerus, Terengganu, Malaysia; ^2^Advanced Nano Materials Research Group (ANOMA), Ionic State Analysis (ISA) Laboratory, Universiti Malaysia Terengganu, 21030 Kuala Nerus, Terengganu, Malaysia; ^3^Biological Security and Sustainability (BIOSES) Research Group, Faculty of Science and Marine Environment, Universiti Malaysia Terengganu, 21030 Kuala Nerus, Terengganu, Malaysia; ^4^X-Ray Crystallography Unit, School of Physics, Universiti Sains Malaysia, 11800 USM, Pulau Pinang, Malaysia; ^5^Faculty of Science, Universiti Teknologi Malaysia, 81310 Skudai, Johor Bahru, Malaysia

## Abstract

This article presents both experimental and computational study of a new Ni(II) complex, namely, bis{2-(2-trifluoromethylbenzylidene)hydrazine-1-carbothioamido-*κ*^2^N^2^, S}nickel(II) (abbreviate as NiL_2_). The complex was synthesized and well characterized using various spectroscopic methods. The single X-ray crystallographic study revealed a distorted square planar geometry around Ni(II) metal ion centre in which the angles deviated from ideal 90° with a maximum value of 6.57° occupied by nitrogen and sulphur donor atoms. The theoretical bond lengths and angles for the NiL_2_ complex were obtained by using the B3LYP level of density function theory (DFT) with LANL2DZ/6-311G (*d*, *p*) basis sets. These results showed very good agreement with the experimental X-ray values. The electrophilicity index (*ω* = 50.233 eV) shows that the NiL_2_ complex is a very strong electrophile. In addition, strong F⋯H/H⋯F interactions with 28.5% of the total Hirshfeld surface analyses in NiL_2_ were obtained indicating that the complex could bind with protein effectively. Furthermore, the new NiL_2_ complex was docked with plasma retinol-binding protein 4 (RBP4) (PDB id: 5NU7), which implied that the NiL_2_ complex bound to Tyrosine 133 and Aspartate 102 amino acids via N-H intermolecular hydrogen bonds.

## 1. Introduction

Recent interest in the chemistry of thiosemicarbazone ligands arises mainly from the potential from both azomethine nitrogen and thiolate sulphur donor atoms with variance coordination modes of either monodentate [1], bidentate [2], or tridentate [3]. This variance can be performed by introducing different substituents in order to form a selection of mononuclear [4] and polynuclear [5] complexes.

The versatility of thiosemicarbazone derivatives and its metal complexes allows for the design and development of bioactive compounds, including anticancer [6], antioxidant [7], and antibacterial [8]. (*E*)-2-(1-(3-Bromophenyl)ethylidene)hydrazine-1-carbothioamide molecule shows high potential in behaving as antimalarial agents [9]. Due to these reasons, their structural details are considered useful for structure activity relationships (SAR) design for future applications.

In continuation of our research to develop coordination chemistry of thiosemicarbazones and their transition metal complexes [10, 11], a new Ni(II) complex, namely, bis{2-(2-trifluoromethylbenzylidene)hydrazine-1-carbothioamido-*κ*^2^N^2^, S} nickel(II), NiL_2_ containing (trifluoromethyl)benzene, and thiosemicarbazone moieties have been synthesized, characterized, and computationally optimized using B3LYP level of density function theory (DFT) with LANL2DZ/6-311G (*d*, *p*) basis sets. The experimental X-ray crystallographic structure of the Ni(II) complex also has been correlated with the corresponding structure optimized at DFT/B3LYP/LANL2DZ/6-311G (*d*, *p*) level. In addition, Hirshfeld surface analysis was also used to interpret intermolecular interactions in the NiL_2_ complex by visual representations whereas molecular docking was studied to know the receptor-amino acid interactions, to predict the important functional groups or atoms in the complex.

## 2. Materials and Methods

### 2.1. General Procedure

All the chemicals were purchased from Aldrich, R&M, and HmbG and used without further purification. Elemental analysis was performed with a CHNS-O Flashea Siri 112 Analyzer. Magnetic measurements were carried out on a Johnson Matthey Mark I MSB magnetic susceptibility balance model MKIC using Gouy's method. The molar conductance of freshly prepared 1.0 × 10^−3^ M in DMSO solutions was measured for the NiL_2_ complex using Jenway 4320 conductivity meter. Electronic spectra were recorded on Shimadzu UV-1800 UV spectrophotometer and the samples were prepared with 1.0 × 10^−5^ M in DMSO solutions.

### 2.2. Synthesis of NiL_2_

**Scheme 1:**
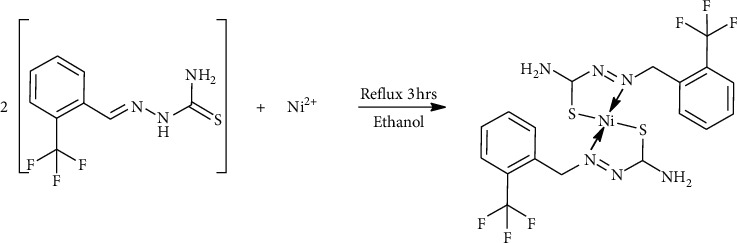
Synthesis of NiL_2_ complex.

A solution of 20 mL hot ethanol of nickel(II) acetate tetrahydrate, Ni(Ac)_2_.4H_2_O (0.07 g, 0.3 mmol) was added to 30 mL hot solution of 1-(2-trifluoromethylbenzylidene)thiosemicarbazide (0.33 g, 0.6 mmol) in a ratio of 1 : 2, respectively. The mixture was heated under reflux for 3 hours. The brown precipitate formed was filtered, washed with cold ethanol, and kept in a desiccator. Suitable single crystals of the NiL_2_ complex were obtained from a methanol : DMF mixture solution through the vapor diffusion method. The chemical equation for the reaction is shown in Scheme 1. Yield 68.28%. Melting point 246.72°C. Analysis calculated for C_18_H_16_N_6_S_2_F_2_Ni: C, 39.88; H, 2.97; N, 15.50; S, 11.83. Found: C, 40.66; H, 3.29; N, 16.14; S, 13.61%. *λ*_max_ (nm): 269 n⟶*π*^*∗*^, 328 (LMCT transition), ∼450 (^1^A_1_g⟶^1^A_2_g). Molar conductance: 1.37 Ω^−1^ cm^2^ mol^−1^. *µ*_eff_ (B.M.): 0.0.

### 2.3. X-Ray Diffraction Studies

A single-crystal X-ray diffraction (SCXRD) study of NiL_2_ was performed on Bruker SMART Apex II Duo CCD area-detector diffractometers using MoK*α* radiation (*λ* = 0.71073 Å). The data collection was performed by APEX2 software [12], whereas the cell refinement and data reduction were performed by SAINT software [12]. The crystallographic structure was solved by Direct Method using SHELXTL [13] and further refined by full-matrix least squares technique on F^2^ using anisotropic displacement parameters by SHELXTL [13]. Absorption correction was applied to the final crystal data using the SADABS software [12]. All geometrical calculations were carried out using the program PLATON [14]. The molecular graphics were drawn using SHELXTL [13]. All the hydrogen atoms were positioned geometrically (C—H = 0.93 Å) and refined using riding model *U*_iso_(H) = 1.2 *U*_eq_(C) which means the isotropic displacement parameters are set to 1.2(C) times the equivalent isotropic U values of the parent carbon atoms. Additionally, the N-bound H atoms were located in a difference Fourier map and freely refined (N—H = 0.86 Å). Selected crystal structure parameters are listed in Table 1.

### 2.4. Computational Details

This study reports computational studies on NiL_2_ complex. All calculations were performed by Gaussian 16 using high performance computer (HPC) provided by CICT, UTM along with Gauss View 6.0 for visualizations. The geometries were fully optimized without any constraint on every bond length, bond angle, and dihedral angle. Geometry optimizations were conducted using the unrestricted DFT method at the level of B3LYP/LANL2DZ/6-311G (*d*, *p*) (B3LYP/GENECP) with the keyword “OPT”. The highest occupied molecular orbital (HOMO) and the lowest unoccupied molecular orbital (LUMO) were also performed under the same basic set. The reactivity descriptors that include energy gap (Δ*E*_gap_), hardness (*η*), softness (*S*), global electronegativity (*χ*), and electrophilicity (*ω*) have also been computed by the same approach as from our previous work [15].

### 2.5. Molecular Docking

The crystal structure of plasma retinol-binding protein (RBP4) was obtained from the RSCB protein database (PDB ID:5NU7). The 5NU7 structure was selected due to high 3D crystal structure resolution at 1.5 Å. The active binding site of RBP4 was predicted using 3DLigandSite (http://www.sbg.bio.ic.ac.uk/∼3dligandsite/) [16]. Docking analysis between NiL_2_ complex and RBP4 was using UCSF Chimera version 1.14 [17], AutoDock Vina [18], and LigPlot + v.1.4 [19]. Methodically, AutoDock Vina used the Broyden–Fletcher–Goldfarb–Shanno algorithm for molecular docking. The docking coordinate (grid box) were determined based on the result from 3DLigandSite, presence of pocket structure, and location of retinol in the RBP4 crystal structure.

## 3. Results and Discussion

**Figure 1:**
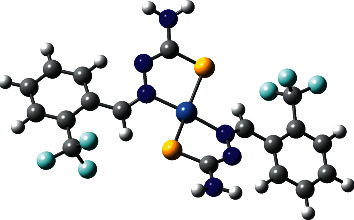
Optimized molecular structure of NiL_2_ complex.

Bis{2-(2-trifluoromethylbenzylidene)hydrazine-1-carbothioamido-*κ*^2^N^2^, S}nickel(II), NiL_2_ complex, was synthesized according to our reported procedures in [10], however with different reactants. Selected experimental and theoretical geometric parameters optimized of NiL_2_ complex structure are shown in Table 2. The molecular structure of the NiL_2_ complex obtained empirically was compared with theoretical calculation via DFT (Figure 1). Percentage of deviation between bond lengths and bond angles for NiL_2_ complex was calculated using equation (1). From Table 2, the average deviation percentage for both bond length and bond angles is at a low value (1.94% and 1.14%) indicating that experimental and calculation work is in good agreement. This is further proven by the statistical correlation graph that shows *R*^*2*^ values of 0.99849 for bond length (Figure S1) and 0.99384 for bond angle (Figure S2). However, there is a slight difference in values from experimental crystallographic data which might be due to the theoretical results obtained for isolated complex in the gaseous phase, whereas the experimental results obtained for both intra- and interlinked complexes in the solid phase similar to the previous report [20]:

(1) $$\begin{array}{c} \text{Percentage deviation}=\frac{\text{calculated value}-\text{experimental value}}{\text{experimental value}}\times 100\%. \end{array}$$

### 3.1. Molecular Structure Studies

**Figure 2:**
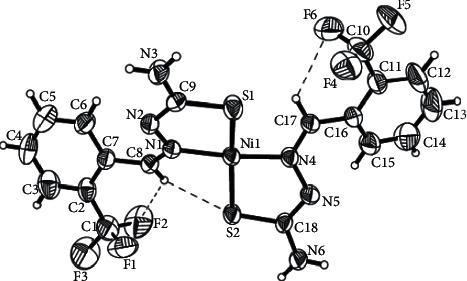
Molecular structure of NiL_2_ complex (50% probability ellipsoids).

X-ray crystal study of structure Bis{2-(2-trifluoromethylbenzylidene)hydrazine-1-carbothioamido-*κ*^2^N^2^, S}nickel(II), (NiL_2_) complex, C_18_H_16_F_6_N_6_S_2_Ni with molecular weight *M* = 553.20 gmol^−1^, showed the NiL_2_ complex crystallized in the orthorhombic system with a space group of *Iba2*. The unit cell dimensions are *a* = 16.889(3)Å, *b* = 15.777(3) Å, *c* = 15.777 Å, and *α* = *β* = *γ* = 90°. Important bond lengths and bond angles are given in Table 2.  Figure 2 shows the molecular structure and atom numbering of the NiL_2_ complex, with thermal ellipsoids drawn at the 50% probability level.

The NiL_2_ complex is a bis-chelate complex of Ni(II) with two L^¯^ ligands acting as bidentate chelate units (Figure 2). The distortion from square planar geometry is mainly due to the S1‒Ni1‒N1, S2‒Ni1‒N1, S2‒Ni1‒N4, and S1‒Ni‒N4 bond angles of 86.70(10), 96.57(10), 84.96(9), and 94.56(10)°, respectively, which differs from the ideal 90° by a maximum value of 6.57°. Two ligand units, placed a bit curved to each other with twisted angles between the planes of the two coordinated ligands, are 25.03(14)° with maximum r.m.s deviation of 0.159(3) A˚ for N4 atom.

The pattern of bond length within the previous 1-(2-trifluoromethylbenzylidene)thiosemicarbazide ligand reported in [21] clearly indicates that the molecule is present in the thioamide form with both C‒N and C=S bonds length of 1.343 (6)Å and 1.699 (4)Å, respectively, consistently with the location of the H atom bonded to N2 atom. However, deprotonated for N2 atom in the present NiL_2_ complex was observed. Data of Table 2 show that, in the Ni(II) complex, there is a shortening and lengthening of both C9-N2 and C9=S1 bonds with 1.303(6) Å and 1.717(4) Å, respectively. Thus, a tautomeric switch from thione to thiol form is postulate.

**Figure 3:**
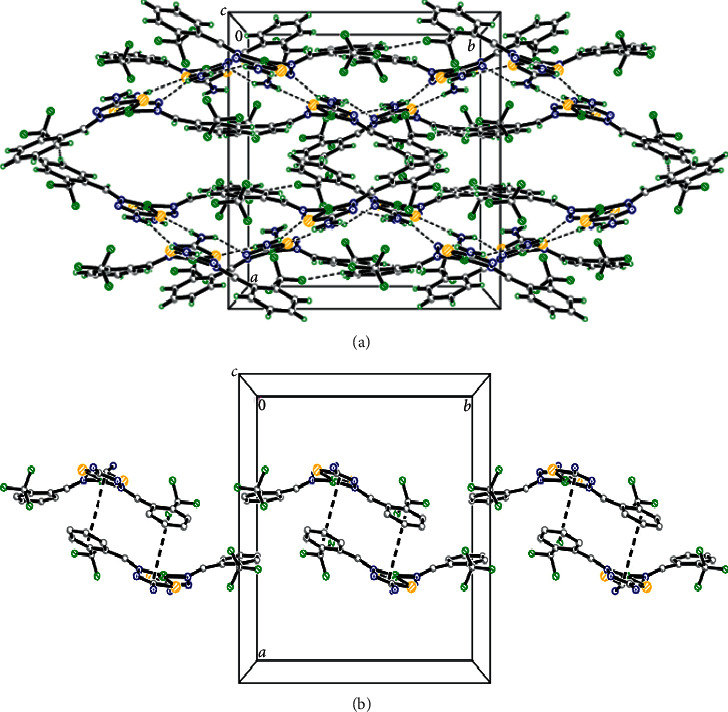
(a) Molecular packing diagram of NiL2 complex, showing molecules connected by intermolecular hydrogen bonds (dashed lines). (b) *π–π* interaction in NiL2 complex.

The molecular packing of NiL_2_ complex is mainly linked by three strong C8-H8A^…^S2, C8-H8A^...^F2, and C17-H17A^…^F6 intramolecular hydrogen bonds listed in Table 3, forming one pseudo-five and two pseudo-six membered graph set motifs. In the crystal structure, the NiL_2_ complex is interconnected through N3‒H3B^...^S2, N3‒H3C^…^N5, N6‒H6B^...^S1, N6‒H6C^...^N2, C5‒H5A^...^F2, and C12‒H12A^...^F3 hydrogen bonds forming a three-dimensional architecture (Figure 3(a)). The molecules are further stabilized by weak *Cg*1^…^*Cg*3 interactions (*Cg1* and *Cg3* are the centroids of Ni1/S1/C9/N2/N1 and C2/C3/C4/C5/C6/C7, resp.) with the contact distance of 3.785(3) Å (symmetry code: 11 − *x*, 1 − *y*, *z*), forming one-dimensional dimeric wave-like parallel to *b-*axis (Figure 3(b)).

### 3.2. UV-Vis Spectroscopy

**Figure 4:**
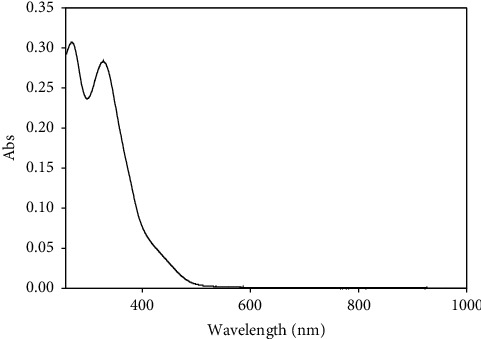
UV-Vis spectrum of NiL_2_ complex.

The UV-Vis spectrum (Figure 4) of the NiL_2_ complex, bis{2-(2-trifluoromethylbenzylidene)hydrazine-1-carbothioamido-*κ*^2^N^2^, S}nickel(II), exhibits two bands at *λ*_max_ = 269 nm and 328 nm, which can be assigned to the *π*⟶*π* transition of the conjugated phenyl ring and *n⟶π*^*∗*^ intraligand charge transfer (ILCT) transition of the C=S and CN chromophore in ligand molecule [22]. In addition, a shoulder band which appeared at ∼450 nm in NiL_2_ complex can be assigned to ^1^A_1_g⟶^1^A_2_g transition. This band is well correlated with the previous study of the square planar NiL_2_ complex [22]. To further support, the magnetic moment of the NiL_2_ complex was shown of value 0 B.M, which is one of the main criteria for square planar geometry. [21] whereas low molar conductance with 1.37 Ω^−1 ^cm^2^ mol^−1^ showed the absence of acetate ion and nonelectrolytes in DMSO solution [23].

### 3.3. Frontier Molecular Orbitals Studies

The highest occupied molecular orbital (HOMO) and the lowest unoccupied molecular orbital (LUMO) are frequently studied in order to impart key information regarding the electron-donor and electron-acceptor character of the complexes which shall lead to the interpretation of the charge transfer process. The lower energy of the HOMO indicates the lower ability as an electron-donor, resulting in higher energy of LUMO and higher resistance to accept electron. This allows elucidation of chemical stability by observing the difference in energy between HOMO and LUMO (*E*_gap_). While large *E*_gap_ is preferred for the high stability of complexes with respect to chemical reactions, low *E*_gap_ is well sought by the researchers in relating to chemical reactivity in applications such as antibacterial studies due to the ability to encounter efficient charge transfer interactions.

In addition, this study can also explain the chemical concept of chemical softness and hardness. With small *E*_gap_, the complexes are considered as “soft” base due to the high energy of HOMO and thus enhance the interaction with the LUMO of soft acids. Other than that, indices such as electron affinity and ionization potential are also commonly interconnected with the studies of HOMO and LUMO energies in pursuing a better grasp of how complexes theoretically behave, chemicalwise.

**Figure 5:**
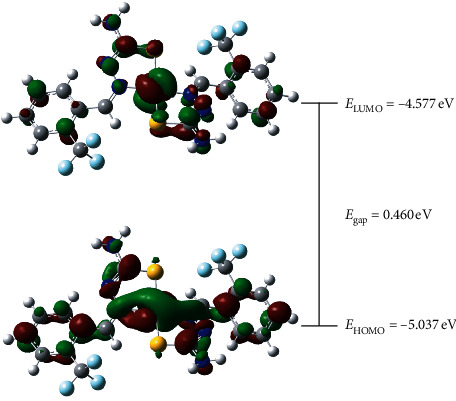
HOMO-LUMO surfaces and energy gap for NiL_2_ complex.

As can be seen in Figure 5, the electron density of the NiL_2_ complex is mainly distributed over the nitrogen, sulphur, and Ni atoms for both HOMO and LUMO. The calculated values of reactivity descriptors parameters are summarized in Table 4. The given low energy gap (0.460 eV) thus indicates high reactivity of the complex due to ease of charge transfer process [24]. The high value of softness (2.174) or the low value of hardness (0.230) indicates lower energy is needed for electron transition from HOMO to LUMO which means that the complex is susceptible to deform and ready to interact with other nucleophilic active site such as amino acid. This is confirmed by the calculation of relatively high value of electrophilic, *ω* (50.233 eV) as compared to other work by our group of similar ligand isomer, namely, (*Z*)-1-[4-(trifluoromethyl)benzylidene]thiosemicarbazide with electrophilic (*ω*) value of 1.8073 eV [25].

### 3.4. Hirshfeld Analysis

**Figure 6:**
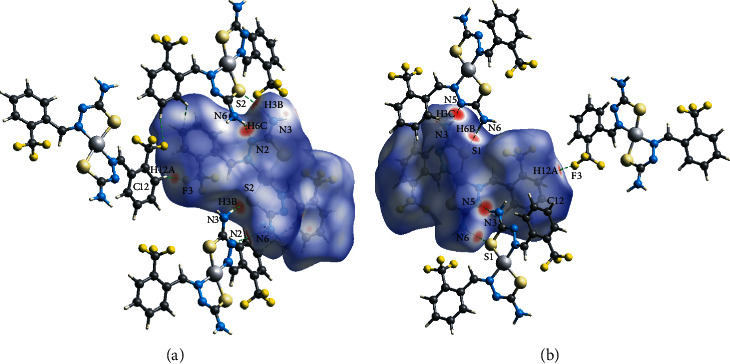
Neighbouring molecules associated with intermolecular hydrogen bonding on a *d*_norm_ at the (a) back and (b) front views of NiL_2_ complex.

Hirshfeld surface analysis has been carried out to illustrate the interactions of the crystal structure and their 2D fingerprint plots were established using CrystalExplorer3.1 software [26]. The Hirshfeld surfaces for *d*_norm_ were obtained and generated as a transparent surface to allow visualization of the molecular structure. The d_norm_ (−0.337 to 1.450) Å mapping of the Hirshfeld surface (Figure 6) exemplified several red spots in various sizes and intensities. The red spots remarked on the NiL_2_ complex showed the dominant interactions involving the donor and acceptor. The C–H⋯F, N–H⋯N and N–H⋯S contacts are present in the studied NiL_2_ complex. As shown in Figure 6, the interactions at the NiL_2_ complex backbone between the hydrogen of the amine and its adjacent sulphur atom (N3–H3B⋯S2) and nitrogen atom (N6–H6C⋯N2) form a dimeric arrangement in the crystal packing. Also, there is an additional red spot representing the intermolecular hydrogen bonding of the C12–H12A···F3 due to the side by side arrangement of the neighbouring complexes in the crystal packing. Besides, from the other side view of the NiL_2_ complex, the intense red spots revealed the intermolecular hydrogen bonding of N3–H3A···N5 and N6–H6B⋯S1 interactions of the NiL_2_ complex (Figure 6). These two interactions between the adjacent NiL_2_ complexes are raised due to their dimeric arrangement in the unit cell packing.

**Figure 7:**
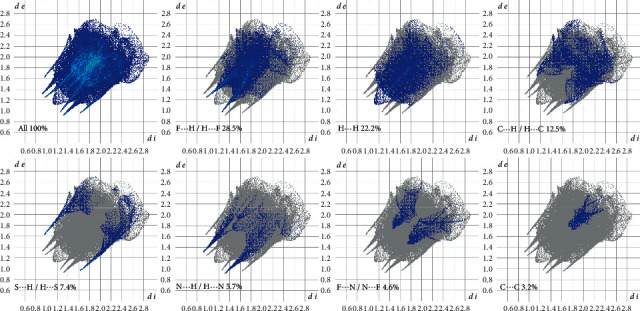
Selected fingerprint plots of the intermolecular interactions showing the percentage contributions to the total Hirshfeld surface.

The fingerprint plots indicate the percentage contributions of the various intermolecular contacts (Figure 7). In order to highlight all the interactions involved in the crystal packing, each fingerprint plot was divided into the specific pairs of atom-types contributions, such as H⋯F, H⋯H, H⋯C, H⋯S, H⋯N, C⋯C, and other. The blue coloured represents the assigned reciprocal contacts, while the grey shadow denotes the outline of the original fingerprint plots [27]. *d*_*e*_ and *d*_*i*_ are the distances from the Hirshfeld surface to the nearest atoms outside and inside the surface [28].

The F⋯H/H⋯F contacts appeared as the largest contribution to the Hirshfeld surface (28.5%). Their two symmetrical narrow spikes of *d*_*e*_ + *d*_*i*_ 2.20 Å proved the presence of the intermolecular C–H⋯F interactions of the NiL_2_ complex. Furthermore, the characteristic spikes representing the shortest H⋯H contacts contributed as the second-largest fingerprint plot to the Hirshfeld surface (22.2%) with a high concentration in the middle region as shown in light blue at *d*_*e*_ + *d*_i_ 2.25 Å. The contribution of the C⋯H/H⋯C (12.5%) is indicated by a pair of peaks at *d*_*e*_ + *d*_*i*_ 2.80 Å. In Addition, the spikes of S⋯H/H⋯S and N⋯H/H⋯N contacts showed 7.4% and 5.7% contribution, respectively, which correspond to the presence of N–H⋯S interactions. The sharpest point in H⋯S featured a closer contact of *d*_*e*_ + *d*_*i*_ 2.60 Å, while *d*_*e*_ + *d*_*i*_ 2.20 Å for N⋯H contacts, respectively. Moreover, the F⋯N/N⋯F contacts showed a 4.6% contribution illustrated by a butterfly fingerprint plot with *d*_*e*_ + *d*_*i*_ 3.20 Å. The C⋯C contacts usually refer to *π–π* staking interaction [29]. In this NiL_2_ complex, C⋯C contacts contributed 3.2% of the Hirshfeld surface with the sum of *d*_*e*_ and *d*_*i*_ being approximately 3.5 Å. There is also a negligible amount of other contacts contribution (C⋯F, C⋯N, F⋯S, C⋯S, and Ni⋯F), with less than 2% in the compound. Thus, their contacts are almost insignificant to discuss.

### 3.5. Molecular Docking

**Figure 8:**
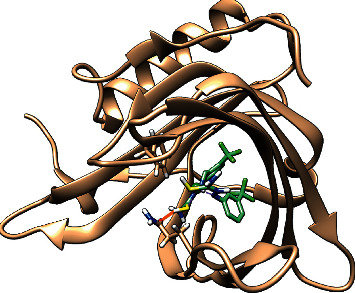
Molecular docking studies of NiL_2_ complex at the active site of RBP4.

**Figure 9:**
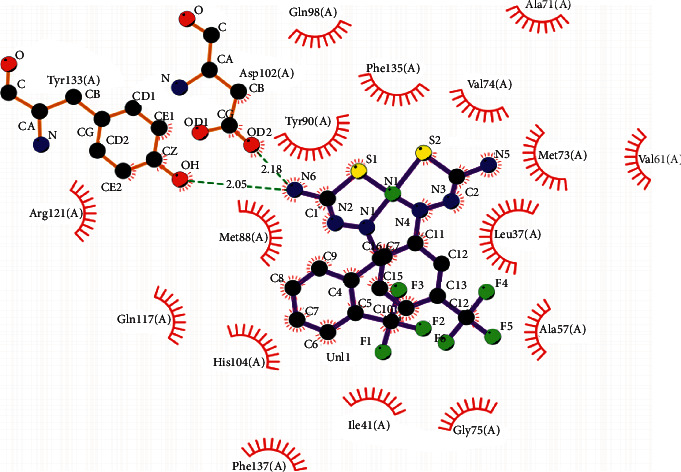
Intermolecular hydrogen bonds of NiL_2_ complex with both Tyrosine 133 and Aspartate 102 at the active site of RBP4.

Docking analysis was conducted to investigate the possibility of molecular interaction of NiL_2_ complex with biologically important proteins. Previous studies have shown the importance of docking analysis for a synthetic compound such as thiosemicarbazide and terphenyl derivatives [30, 31]. Interaction of NiL_2_ complex with plasma retinol-binding protein 4 (RBP4) (PDB id: 5NU7) was investigated to understand its potency. Physiologically, RBP4 acts as a transporter for retinol [32]. The NiL_2_ complex docked inside the RBP4 active binding site which is at the same site as the retinol. A total of ten possible 3D docking orientations of NiL_2_ complex inside RB4 active binding site are seen, the highest docking rank with the lowest energy (kcal/mol) as shown in Figure 8. Binding affinity is calculated as −3.3 Kcal mol^−1^ for NiL_2_ complex and closer to retinol with −5.5 Kcal mol^−1^ (Table 5). According to these results, the most effective intermolecular hydrogen bonds are observed between NiL_2_ complex through N-H atoms and Tyrosine 133 (2.05 Å) or Aspartate 102 (2.18 Å) in the active binding site (Figure 9). However, the N-H intramolecular hydrogen interaction is contrasted with Hirshfeld surface analysis, where F-H interaction is the most dominant (28.5%). It is due to the rotation around both Ni-N and Ni-S bonds in NiL_2_ complex for insertion into the RBP4 active site. Similar formations of both antisymmetrical (anti) and symmetrical (syn) isomers of the Pd(II) and Pt(II) complexes have been previously reported [33, 34]. Therefore, the present NiL_2_ complex has the potential to be a competitive substrate for retinol that is able to bind at the same active binding site of RBP4. Due to the high number of amino acids interacting with the NiL_2_ complex (via hydrophobic interaction and hydrogen bonds), the release of NiL_2_ complex from the transporter protein would be slower than the retinol.

## 4. Conclusion

A new complex, bis{2-(2-trifluoromethylbenzylidene)hydrazine-1-carbothioamido-*κ*^2^N^2^, S}nickel(II), (NiL_2_) was prepared and its structure was characterized by elemental analysis, molar conductance, magnetic susceptibility, and UV-Vis. The structure has been further confirmed by the single X-ray crystallographic which showed a distorted square planar geometry. In addition, the structure of the synthesized NiL_2_ complex is stabilized by *π–π*, inter- and intramolecular interactions. The Hirshfeld surface analysis has confirmed the presence of several interactions with C–H⋯F interactions being the most important features of crystal packing. The paramount findings by the HOMO-LUMO energy gap proven the efficiency of this complex to have charge transfer interactions within the molecule due to the small *E*_gap_. This suggested the facile electrons transfer from the NiL_2_ donor orbital to the amino acid acceptor, Finally, molecular docking modelling is illustrated between NiL_2_ complex and plasma retinol-binding protein 4 (RBP4) (PDB id: 5NU7) active site. NiL_2_ has interacted with both Tyrosine 133 and Aspartate 102 amino acids through N-H hydrogen bonds.

## Figures and Tables

**Table 1:** Crystal data and structure refinement for NiL_2_.

CCDC	2023702
Empirical formula	C_18_H_16_F_6_N_6_S_2_Ni
Formula weight	553.20
Temperature	296(2) K
Wavelength	0.71073 Å
Crystal system	*Iba2*
Space group	Orthorhombic
Unit cell dimensions	*a* = 16.889(3) Å *α* = 90°
	*b* = 15.777(3) Å *β* = 90°
	*c* = 15.777 Å *γ* = 90°
Volume	4203.7(10) Å^3^
Z	8
Density (calculated)	1.748 Mg/m^3^
Absorption coefficient	1.195 mm^−1^
F(000)	2240
Crystal size	0.35 × 0.22 × 0.10 mm^3^
Theta range for data collection	2.41 to 30.15°
Index ranges	*h* = −23⟶23
	*k* = −22⟶18
	*l* = −21⟶21
Reflections collected	18101
Independent reflections	5305 [*R*_int_ = 0.0691]
Completeness to theta = 30.15°	87.0%
Refinement method	Full-matrix least-squares on F^2^
Data/restraints/parameters	5305/1/298
Goodness-of-fit on F^2^	0.749
Final *R* indices [*I* >2sigma(*I*)]	*R*_1_ = 0.0432, wR_2_ = 0.0652
*R* indices (all data)	*R*_1_ = 0.0934, wR_2_ = 0.0755
Absolute structure parameter	−0.006(13)
Largest diff. peak and hole	0.394 and −0.231 e.Å^3^

**Table 2:** The experimental and optimized bond lengths (Å) and angles (°) for NiL_2_.

Bond lengths				Bond angles			
	Exp.	DFT	Deviation (%)		Exp.	DFT	Deviation (%)
Ni1-S2	2.175(11)	2.253	3.61	S1-Ni1-N1	86.70(10)	85.50	1.38
Ni1-N4	1.888(3)	1.946	3.07	S2-Ni1-N1	96.57(10)	96.00	0.59
Ni1-S1	2.159(12)	2.253	4.36	Ni1-S1-C9	95.78(14)	93.55	2.33
Ni1-N1	1.892(3)	1.946	2.85	S1-C9-N2	123.50(3)	124.7	0.97
S2-C18	1.722(4)	1.745	1.33	N1-N2-C9	112.70(3)	113.7	0.89
N4-N5	1.371(4)	1.366	0.36	Ni1-N1-N2	120.90(2)	120.6	0.25
N5-C18	1.301(6)	1.311	0.76	S1-Ni1-N4	94.56(10)	96.00	1.52
N6-C18	1.333(5)	1.359	1.95	S2-Ni1-N4	84.96(9)	85.50	0.64
S1-C9	1.717(4)	1.745	1.63	Ni1-S2-C18	95.14(13)	93.55	1.67
N1-N2	1.380(4)	1.366	1.01	S2-C18-N5	121.9(3)	124.7	2.30
N2-C9	1.303(6)	1.311	0.61	N4-N5-C18	112.9(3)	113.7	0.71
N3-C9	1.336(5)	1.359	1.72	Ni1-N4- N5	120.1(2)	120.6	0.42

**Table 3:** Hydrogen bonds for NiL_2_ complex.

D-H^...^A	D-H	H^...^A	D^...^A	D-H^…^A
C8-H8A^…^S2	0.93	2.52	3.125(5)	123
C8-H8A^...^F2	0.93	2.31	2.964(6)	127
C17-H17A^…^F6	0.93	2.46	3.072(5)	124
N3-H3B^...^S2^i^	0.86	2.68	3.531(3)	170
N3-H3C^…^N5^ii^	0.86	2.37	3.198(5)	162
N6-H6B^...^S1^iii^	0.86	2.76	3.622(3)	179
N6-H6C^...^N2^iv^	0.86	2.41	3.188(5)	150
C5-H5A^...^F2^v^	0.93	2.35	3.276(6)	175
C12-H12A^...^F3^vi^	0.93	2.47	3.330(7)	155

Symmetry codes: (i) *x*, 1 − *y*, 1/2 + *z*; (ii) 1/2 − *x*, 1/2 − *y*, 1/2 + *z*; (iii) 1/2 − *x*, 1/2 − *y*, −1/2 + ; (iv) *x*, 1 − *y*, −1/2 + *z*; (v) 1 − *x*, *y*, 1/2 + *z*; (vi) *x*, −1 + *y*, *z*.

**Table 4:** Reactivity descriptors (in eV) of NiL_2_ complex, computed at B3LYP/LANL2DZ/6-311G (*d*, *p*) level of theory.

Parameter	
*E*_HOMO_	−5.037
*E*_LUMO_	−4.577
Energy gap, Δ*E*_GAP_ = *E*_LUMO_ − *E*_HOMO_	0.460
Ionisation potential, *I* = −E_HOMO_	5.037
Electron affinity, A = −E_LUMO_	4.577
Hardness, *η* = *E*_LUMO_ − *E*_HOMO_ 2	0.230
Softness, *S* = 1/2*η*	2.174
Chemical potential, *μ* = − (I + *A*)/2	−4.807
Absolute electronegativity, *χ* = (I + *A*)/2	4.807
Electrophilicity, *ω* = *χ*^2^/2*η*	50.233

**Table 5:** Binding affinities (Kcal mol^−1^) and number hydrogen bonds (*H*_bonds_) from Autodock analysis of retinol and NiL_2_ complex.

Substrate	Energy (Kcal mol^−1^)	*H*_bonds_
Retinol	−5.5	0
NiL_2_	−3.3	1

## Data Availability

Crystallographic data for the structure reported in this study is deposited at the Cambridge Crystallographic Data Centre under the CCDC no. 2023702. These data can be obtained free of charge via the Cambridge Crystallographic Data Centre, 12 Union Road, Cambridge CB2 1EZ, UK; fax: (+44) 1223-336-033; e-mail through deposit@ccdc.cam.ac.uk.

## Supplementary Materials

Supplementary Materials Supplementary data include CIF file of the most important compounds described in this article. CCDC no. 2023702 contains supplementary crystallographic data for NiL_2._.
