# Supplementary material for: Synthesis, X-Ray Structure, Hirshfeld Surface Analysis, DFT Calculations, and Molecular Docking Studies of Nickel(II) Complex with Thiosemicarbazone Derivative
Source: Bioinorg Chem Appl. 2021 May 26;2021:5536902. doi: 10.1155/2021/5536902 (PMC8175184; doi:10.1155/2021/5536902)
Supplement: Supplementary Materials — Supplementary data include CIF file of the most important compounds described in this article. CCDC no. 2023702 contains supplementary crystallographic data for NiL2.. [file 5536902.f1.docx]

**Synthesis, X-ray Structure, Hirshfeld Surface Analysis, DFT Calculations and Molecular Docking studies of nickel(II) complex with thiosemicarbazone derivative**

**Uwaisulqarni M. Osman^1,2^, Sharmili Silvarajoo^1^, Muhamad Fairus Noor Hassim^3^, Suhana Arshad^4^_,_ Ainizatul Husna Anizaim^4^, Fazira Ilyana Abdul Razak^5^**

^1^Faculty of Science and Marine Environment, Universiti Malaysia Terengganu,

21030 Kuala Nerus, Terengganu, Malaysia

^2^Advanced Nano Materials Research Group (ANOMA), Ionic State Analysis (ISA) Laboratory, Universiti Malaysia Terengganu, 21030 Kuala Nerus, Terengganu, Malaysia

^3^Biological Security and Sustainability (BIOSES) research group, Faculty of Science and Marine Environment, Universiti Malaysia Terengganu, 21030 Kuala Nerus, Terengganu, Malaysia

^4^X-ray Crystallography Unit, School of Physics, Universiti Sains Malaysia, 11800, USM Pulau Pinang, Malaysia

^5^Faculty of Science, Universiti Teknologi Malaysia, 81310, Skudai, Johor Bahru, Malaysia

Correspondence should be addressed to Uwaisulqarni M. Osman; [uwais@umt.edu.my](mailto:uwais@umt.edu.my)

**Figure S1.** Correlation plot for calculated and experimental bond lengths of NiL_2_ complex.

**Figure S2.** Correlation plot for calculated and experimental bond angles of NiL_2_ complex.
